# Triggering the Personalization Backfire Effect: The Moderating Role of Situational Privacy Concern

**DOI:** 10.3390/bs15101323

**Published:** 2025-09-26

**Authors:** Hyeongseok Kim, Seunghee Han

**Affiliations:** Department of Business Administration, Chung-Ang University, Seoul 06974, Republic of Korea

**Keywords:** personalized marketing, privacy concern, personalization–privacy paradox

## Abstract

Personalized marketing presents a powerful but delicate strategy, as its benefits can be negated by rising consumer privacy concerns. To illuminate this tension, this study investigates what causes personalization to fail, focusing on the interaction between the level of message personalization and situationally activated privacy concerns. We conducted a 3 (Message Personalization: Low, Medium, High) × 2 (Situational Privacy Concern: Low, High) between-subjects experiment with 360 participants. These personalization levels were designed as an ecologically valid “intrusiveness ladder,” moving from a generic message to one using contextual data and to one using personally identifiable information (PII). Situational privacy concern was experimentally induced using a news article prime, after which participants were exposed to one of the marketing messages. The results revealed a significant interaction effect that demonstrates a critical “tipping point.” In the low privacy concern condition, increasing personalization boosted purchase intention. Conversely, when privacy concerns were activated, a ‘backfire effect’ occurred: highly intrusive, PII-based personalization was no more effective than a generic message and was significantly less effective than moderate, contextual personalization. Our findings provide causal evidence for the moderating role of situational privacy concern, demonstrating that activating this state is a key condition that triggers a non-linear consumer response. Practitioners must calibrate the level of data intrusiveness, as the most aggressive tactics can be counterproductive when consumer privacy sensitivities are high.

## 1. Introduction

In the age of artificial intelligence (AI) and big data, personalized marketing has evolved from a novel strategy into a ubiquitous and sophisticated practice. Firms now deploy advanced algorithms to analyze vast datasets, from browsing history to real-time location, shaping a hyper-relevant world where consumer needs are anticipated and met with unprecedented efficiency (Frank et al., 2022). This shift has profound implications, particularly in areas like AI-assisted selling, where data collection and personalization are central to the business model (Liu et al., 2025).

However, this rise in personalization is not without cost, as it simultaneously fuels significant consumer privacy concerns. This tension is at the heart of the “personalization–privacy paradox” (Spiekermann et al., 2001), a concept that has gained renewed urgency in the AI era. While consumers often benefit from relevance, advanced personalization can lead them to believe the firm’s tactics are intrusive or manipulative, which in turn reduces purchase intent (Liu et al., 2025). This is a tangible business concern; for instance, the 2023 survey in South Korea (Personal Information Protection Commission & Korea Internet & Security Agency, 2024) found that over 78% of citizens are concerned about the misuse of their personal in-formation.

Much of the current research rightly focuses on the technological drivers of this paradox. Yet, a critical gap remains in understanding the psychological conditions that determine when personalization is perceived as helpful versus harmful. While emerging research suggests privacy concern is not a stable trait but a context-dependent state that can be momentarily activated (Kim et al., 2018), and other work has observed a non-linear “tipping point” where more personalization backfires (Sun et al., 2025), the causal link between these two phenomena remains untested. The critical question is not simply if personalization works, but under what psychological conditions it succeeds or fails.

This study addresses this gap by experimentally investigating the interaction between situationally induced privacy concern and the level of message personalization. Our primary contribution is to experimentally integrate these two streams of research. We provide a causal test of a situational model of privacy concern, seeking to demonstrate that activating this transient state is a key boundary condition that causes the non-linear, backfire effect to manifest. By manipulating participants’ momentary privacy concerns and exposing them to marketing messages with varying levels of data intrusiveness, our research provides a crucial, process-oriented explanation for when and why the promise of personalization fails.

## 2. Literature Review and Hypothesis Development

### 2.1. The Evolution of Personalized Marketing

Personalization involves strategically suggesting products or services that are expected to best fit a customer’s situation based on their individual data (Baek & Morimoto, 2012; White et al., 2008). Recent advances in artificial intelligence (AI) have further accelerated this practice, leveraging sophisticated algorithms to tailor interactions by collecting both explicit information provided by consumers as well as implicit information inferred from behavioral traces such as browsing history and past purchases to increase the perceived relevance (Mehmood et al., 2024; Tucker, 2014).

### 2.2. The Double-Edge of Personalized Marketing

The widespread adoption of personalized marketing is driven by robust evidence of its positive impacts. A recent meta-analysis of experimental studies concludes that personalized ads generally outperform generic ones across attitudinal and behavioral metrics mainly through increased personal relevance (Yeo et al., 2025). At the same time, recent work continues to document that the same cues that signal relevance can also activate privacy concerns and “creepiness,” which undermines effectiveness via lower trust, message rejection, and avoidance behaviors (Baek & Morimoto, 2012; Boerman et al., 2021; De Keyzer et al., 2024; White et al., 2008). The very specificity that makes personalization effective can also trigger consumer concerns about privacy invasion. Public sentiments mirrors theses tensions: more than half of U.S. adults report that personalized ads “creep them out” (YouGov, 2025).

Together, these findings crystallize a paradox: personalization often works because it heightens relevance, yet the very relevance can trigger avoidance and reactance (Bleier & Eisenbeiss, 2015; De Keyzer et al., 2022). This suggests a tipping point dynamic, with some evidence pointing to an inverted-U relationship where moderate personalization is optimal (Sun et al., 2025).

### 2.3. The Role and Sensitivity of Personal Information

The efficacy of personalized marketing is contingent on using personal information. Critically, not all personal information carries the same weight. Under the EU General Data Protection Regulation, personal data encompasses any information relating to an identifiable person, with special categories receiving heightened protection (General Data Protection Regulation [GDPR], 2016). Beyond legal categories, consumers systematically rank some attributes as more sensitive than others. The academic literature consistently shows that certain data types—such as health or financial records—are perceived as highly sensitive (Leslie et al., 2018). Moreover, the combination of seemingly innocuous signals can increase identifiability and perceived intrusiveness (Keyzer et al., 2022; Tao et al., 2024). Technical research reinforces this point: even sparse mobility traces or a handful of demographic points can be enough to identify individuals, and thus modern reviews emphasize that de-identification alone is often not sufficient in the big-data era (Gadotti et al., 2024). This highlights the need for marketers to understand that the type and combination of data used are key factors in consumer acceptance or rejection.

Reflecting this, and for clarity in our study, we distinguish between personally identifiable information (PII), which can be directly linked to a person (e.g., name), and non-personally identifiable information (non-PII), which is more anonymous (e.g., coarse location or behavior). This distinction is widely used in practice and policy discussions (Federal Trade Commission [FTC], 2012; General Data Protection Regulation [GDPR], 2016) and tracks consumers’ differential sensitivity to data types.

Building on this framework, we conceptualize personalization not as a simple binary (personalized vs. not) but as a continuum of data intrusiveness. Therefore, we designed three distinct levels for our experiment to represent key points along this continuum. The low-personalization level serves as a control condition, representing a generic message with no personal data. The medium-personalization level utilizes anonymous, non-personally identifiable information (non-PII), such as the consumer’s general location, which provides context without revealing personal identity. Finally, the high-personalization level represents a significantly more intrusive approach by combining explicit personally identifiable information (PII), like the consumer’s name, with their specific behavioral history. This three-level design is critical as it allows us to not only test the baseline effectiveness of personalization but also to identify a potential “tipping point” where increasing data intrusiveness may lead to a non-linear or even negative consumer response.

Before testing for the complex “tipping point” effect described above, it is necessary to first establish the foundational premise of personalized marketing. Given that the primary benefit of personalization is its ability to increase message relevance, we first predict a main effect in line with the literature (Yeo et al., 2025). Specifically, we expect that messages utilizing either contextual (medium-level) or PII-based (high-level) data will be more effective than a generic, non-personalized approach. This leads to our foundational hypothesis:

**Hypothesis** **1:** *Messages personalized with consumer information will lead to a higher purchase intention than non-personalized messages.*

### 2.4. Situational Privacy Concern as a Key Moderator

Privacy concern is an individual’s subjective apprehension about the potential loss of privacy from data collection and use (Dinev & Hart, 2006; Smith et al., 1996). This concept is distinct from objective risk, as it focuses on an individual’s perception of vulnerability.

While early research often treated privacy concern as a stable individual trait, the contemporary view, which this study adopts, emphasizes its dynamic, situational nature. This perspective posits that concern can be momentarily triggered by contextual cues, such as media reports about data breaches (Frank et al., 2022; Kim et al., 2018), functioning as a transient psychological state.

We propose that this momentary state of privacy concern acts as a critical moderator for the effectiveness of personalization. When such concerns are not active, consumers are likely to focus on the utilitarian benefits of personalization, leading to a positive evaluation as proposed in H1. However, when situational cues have made privacy concerns salient, consumers’ focus will shift from convenience to threat, leading to a negative evaluation of intrusive messages (Lee et al., 2025; Li & Shi, 2025; Segijn et al., 2024). This logic leads to our central interaction hypothesis:

**Hypothesis** **2:** *The level of situational privacy concern will moderate the effect of message personalization on purchase intention.*

To specify this interaction, we propose two sub-hypotheses that predict the effect of personalization within each context. In a low-concern state, consumers are expected to focus on the utilitarian benefits of the message. Therefore, we predict a straightforward positive relationship:

**Hypothesis** **2a:** *In a low privacy concern context, a higher level of message personalization will lead to a higher purchase intention.*

Conversely, when privacy concerns are activated, we predict a curvilinear effect, an idea grounded in psychological reactance theory (Brehm, 1966). While moderate personalization may be seen as helpful, highly intrusive personalization using PII can be perceived as surveillance, triggering reactance and leading consumers to reject the message (Bleier & Eisenbeiss, 2015). Recent studies have empirically identified such a curvilinear pattern, where moderate personalization performs best while high personalization backfires (De Keyzer et al., 2022; Sun et al., 2025). We hypothesize that this backfire is specifically triggered by a state of high situational concern. Thus, we hypothesize the following:

**Hypothesis** **2b:** *In a high privacy concern context, the effect of personalization will be curvilinear: moderate personalization will increase purchase intention relative to no personalization, but high personalization will be less effective than moderate personalization.*

## 3. Materials and Methods

### 3.1. Research Design

This study employed a 3 (Message Personalization: High vs. Medium vs. Low) × 2 (Situational Privacy Concern: High vs. Low) between-subjects factorial design. The primary objective was to investigate the interactive effect of message personalization and situationally activated privacy concern on the dependent variable, purchase intention.

### 3.2. Participants and Procedure

A total of 360 participants were recruited in 2023 via an online survey platform in South Korea. The sample consisted of 198 men (55.0%) and 162 women (45.0%), with a mean age of 37.8 years (SD = 8.9).

The experimental procedure began with participants being randomly assigned to one of the six conditions. First, to standardize the context for the subsequent marketing message, all participants were asked to adopt a detailed hypothetical persona. Specifically, they were instructed to assume their name was “Kim Jung-ang,” that they were a regular customer who shopped at the “Homeplus” hypermarket’s Sindorim branch at least twice a month, and that they had purchased 100,000 won worth of daily necessities from that same store one week prior. Next, to manipulate situational privacy concern, they were instructed to read one of two mock newspaper articles. Following this prime, they were exposed to one of three versions of a marketing message from “Homeplus.” Immediately after viewing the message, participants completed the dependent measures, manipulation checks, and control variable questions. Finally, they provided demographic information and were debriefed.

### 3.3. Experimental Stimuli and Manipulations

The first independent variable, situational privacy concern, was manipulated using a news article prime. In the high-concern condition, participants read an article about recent, large-scale data breaches and fines levied against major tech companies for misuse of personal information. In the low-concern (control) condition, participants read a neutral article of similar length and format about a general topic, such as interior design trends. A pretest revealed that the data breach article (M = 6.1, SD = 0.95) was perceived as significantly more concerning than the neutral article (M = 2.3, SD = 1.1), *t*(48) = 14.5, *p* < 0.001.

The second independent variable, message personalization, was operationalized across three advertisement levels. To ensure high external validity, the experimental messages were developed by adapting the real message format and graphics from a leading telecommunication company. They were subsequently vetted by marketing professionals at the firm to confirm they realistically reflected their marketing efforts.

The high-personalization message incorporated both personally identifiable information (PII) and specific behavior data, using the persona’s name, location, recent shopping activity, and the purchase amount to create a highly intrusive message (e.g., Hello, Mr./Ms. Kim Jung-ang. I see you’re near the Homeplus Sindorim branch, which you visited last week. We’re offering you a coupon for up to 20% off, based on the 100,000 won you recently spent at Homeplus!”). The medium-personalization message demonstrated an awareness of the participant’s current location to entice them with a coupon, using only contextual, non-personally identifiable information (e.g., “Hello, customer. We’re offering a coupon for up to 20% off at Homeplus stores within 1km of your current location!”) Finally, the low-personalization (control) message was a generic advertisement with no personal information (e.g., “Hello, customer. We’re offering a coupon for you to receive up to 20% off at Homeplus!”).

In this study, we conceptualize “personalization” as a continuum of data intrusiveness. We acknowledge that our design choice, which moves from contextual cues to PII, introduces a conceptual confound between the quantity and type of data used. This was a deliberate choice to maximize ecological validity. Accordingly, our experiment is designed to test practice-oriented boundary conditions by treating this manipulation as an “intrusiveness ladder,” rather than to isolate a “pure intensity” effect. This approach allows us to test escalating personalization as it commonly occurs in practice.

The full text for all stimuli is provided in Appendix A.

### 3.4. Measures

To measure purchase intention, we used the product purchase intention measurement tool from Gefen and Straub (2004). Participants indicated their level of agreement on a 7-point Likert scale (1 = Not at all, 7 = Very much so) with five items (Cronbach’s Alpha = 0.960): “I think this product is necessary for me,” “I am likely to purchase this product,” “I will continue to purchase this product in the future,” “I want to click the URL in the marketing text message to get more information about the product,” and “I want to continue receiving advertising messages like this one in the future.” Purchase intention was measured as the average of the responses to these five items.

To measure perceived personalization, we adapted items from studies on personalized marketing by Baek and Morimoto (2012) and Xu et al. (2011) to fit our research context. Participants indicated their level of agreement on a 7-point Likert scale (1 = Not at all, 7 = Very much so) with three items (Cronbach’s Alpha = 0.990): “The message provides personalized advertising that fits my needs,” “The message provides more relevant product information tailored to my tastes or personal interests,” and “The message helps me purchase products that are right for me.” The degree of personalization was measured as the average of the responses to these three items. These same items were also used as a manipulation check to confirm that the personalization manipulation was successful.

To measure privacy concern, we adapted an item from Dolnicar and Jordaan’s (2007) study on information privacy in direct marketing. Participants indicated their level of agreement on a 7-point Likert scale (1 = Not at all, 7 = Very much so) with a single item: “I am worried that my personal information might be exposed to risk.”

Next, we measured brand trust and prior experience with the marketing company. Brand trust was measured by adapting an item from Ballester and Munuera-Alemán’s (2001) study on brand trust. Participants indicated their agreement on a 7-point Likert scale (1 = Not at all, 7 = Very much so) with the single item: “Homeplus is a trustworthy company.” Prior experience with the marketing brand was measured numerically by asking about recent visitation (“I have visited Homeplus within the last three months”). Finally, we collected demographic information necessary for statistical analysis. None of these control variables, including brand trust, prior experience, or any demographic factors, had a significant effect on the results of our analysis.

## 4. Results

### 4.1. Manipulation Checks

To verify the effectiveness of the experimental manipulations, a series of checks were conducted for both independent variables: situational privacy concern and message personalization. An independent-samples t-test confirmed that participants in the high-concern condition (M = 6.31, SD = 1.02) reported significantly higher levels of state privacy concern than those in the low-concern control condition (M = 5.34, SD = 1.18), *t*(358) = 8.27, *p* < 0.001, *d* = 0.87. A one-way ANOVA on perceived personalization was also significant, *F*(2, 357) = 97.33, *p* < 0.001, *η*_p_^2^ = 0.35. Post hoc tests showed that the high-personalization message (M = 6.41), medium-personalization message (M = 6.04), and low-personalization message (M = 4.87) were all perceived as significantly different from one another (all *p*s ≤ 0.006). Both manipulations were therefore successful.

### 4.2. Hypothesis Testing

To test for the predicted interaction effect, a 3 (Message Personalization: High, Medium, Low) × 2 (Situational Privacy Concern: High, Low) between-subjects ANOVA was conducted on purchase intention. Descriptive statistics for purchase intention across all six conditions are presented in Table 1. The full results of the 3 × 2 ANOVA are presented in Table 2.

The analysis revealed a significant main effect of message personalization, supporting H1, *F*(2, 354) = 8.32, *p* < 0.001, *η*_p_^2^ = 0.045. It also revealed a significant main effect of privacy concern, *F*(1, 354) = 26.50, *p* < 0.001, *η*_p_^2^ = 0.07. Most importantly, and in support of Hypothesis 2, these main effects were qualified by a significant two-way interaction between message personalization and privacy concern, *F*(2, 354) = 3.21, *p* = 0.042, *η*_p_^2^ = 0.018. This interaction indicates that the effect of message personalization on purchase intention depends on the consumer’s level of situational privacy concern. A post hoc sensitivity analysis indicates that our sample size (N = 360) provided 80% power to detect an effect size of *f* = 0.15 for the interaction, confirming that while the effect is small, it was detectable with our design.

**Figure 1:**
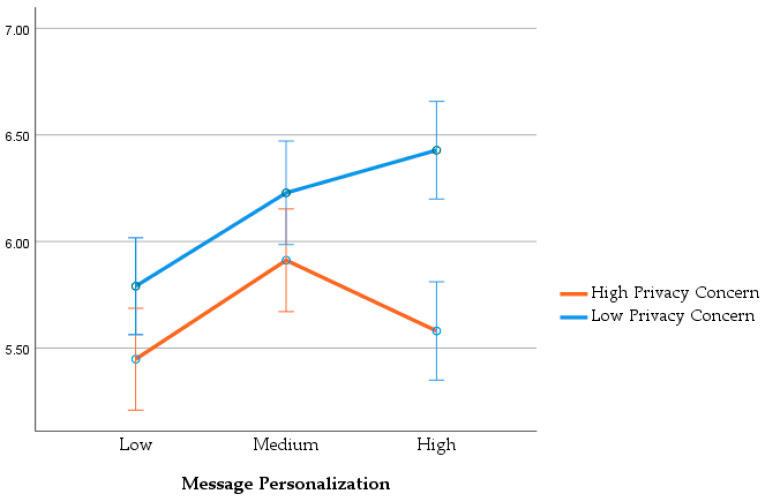
Estimated Marginal Means of Purchase Intention. Error bars represent 95% confidence intervals. Per-cell sample sizes (n) were: Low Concern/Low Person. (64), Low Concern/Med Person. (56), Low Concern/High Person. (63); High Concern/Low Person. (58), High Concern/Med Person. (57), High Concern/High Person. (62).

The nature of this interaction, illustrated in Figure 1, was explored further by analyzing the simple effects for each privacy concern condition, which correspond to Hypotheses 2a and 2b.

In the low privacy concern condition, there was a significant effect of personalization on purchase intention, supporting Hypothesis 2a, *F*(2, 180) = 8.17, *p* < 0.001, η_p_^2^ = 0.083. Post hoc tests showed that both high personalization (M = 6.43) and medium personalization (M = 6.23) led to significantly higher purchase intention than low personalization (M = 5.79; *p* = 0.001 and *p* = 0.03, respectively).

In the high privacy concern condition, there was also a significant effect of personalization on purchase intention, *F*(2, 174) = 3.72, *p* = 0.026, *η*_p_^2^ = 0.041. However, the pattern of results was curvilinear, supporting Hypothesis 2b. Post hoc tests revealed that medium personalization (M = 5.91) led to significantly higher purchase intention than low personalization (M = 5.45, *p* = 0.03). Critically, high personalization (M = 5.58) was not significantly different from the low-personalization control and was directionally lower than medium personalization. This demonstrates a “backfire effect” where the most intrusive message was no more effective than a generic one.

## 5. Discussion

### 5.1. General Discussion

This study examined how message personalization and situational privacy concern jointly shape consumer purchase intention. Consistent with contemporary evidence, we find that personalization generally improves responses relative to non-personalized messages, reinforcing the field’s consensus that, on average, personalized advertising outperforms generic alternatives (Yeo et al., 2025). At the same time, the benefits are conditional: their magnitudes vary by consumers’ momentary mindset. This aligns with recent demonstrations that more personalization is not always better, and that optimal level may be moderate rather than maximal (Sun et al., 2025).

Our baseline results—where both medium (location-based) and high (name/history-based) personalization surpassed a generic control—track with evidence that perceived relevance is critical for personalization’s persuasiveness (An & Ngo, 2025; Yeo et al., 2025). However, the non-significant incremental lift from high (PII-based) over moderate (contextual) personalization suggests diminishing returns even when privacy concern is low. This pattern echoes field and lab studies showing that overly specific or “too tight” targeting can underperform more contextual appeals unless the consumer is far along in the decision process (Bleier & Eisenbeiss, 2015).

Critically, when privacy concern is situationally elevated, our data reveal a backfire: high (PII-based) personalization underperforms moderate personalization and is no better than a generic message. This curvilinear pattern matches new experiments documenting an inverted-U shape relationship between personalization and its effectiveness (Sun et al., 2025; De Keyzer et al., 2022).

### 5.2. Theoretical Implications

Our findings offer two specific theoretical contributions.

First, our study provides causal evidence for a situational view of the personalization–privacy paradox. Building on the conceptual work of Kim et al. (2018), we show experimentally that activating a transient state of privacy concern moderates how consumers evaluate personalization. This dovetails with experiments that induce privacy salience via brief primes and show corresponding shifts in technology/app evaluations. Our contribution is to demonstrate that such state changes directly recalibrate the utility-threat tradeoff in consumer response to advertising, shifting the focus from who is privacy-sensitive to when and why these sensitivities become behaviorally relevant.

Second, we elaborate a non-linear account of personalization effectiveness by specifying a key condition under which it fails. We show that under high situational concern, moving from moderate to high (PII-based) personalization crosses a perceived intrusiveness threshold. While our study did not measure mediating mechanisms, this backfire effect is consistent with plausible theoretical explanations such as psychological reactance, where perceived surveillance threatens consumer autonomy, or heightened perceptions of vulnerability and loss of control. Our work replaces a vague “too creepy” narrative with a predictable interaction between data intrusiveness and situational concern, providing a more precise model for future research to test these underlying mechanisms.

### 5.3. Practical Implications

The findings yield useful insights for anyone designing personalized systems, including marketing practitioners, user experience (UX) designers, and communication strategists. The key takeaway is that personalization strategies must be calibrated with care. An aggressive, “one-size-fits-all” approach that uses the most intrusive data available is risky and can be counterproductive.

System designers and managers should reconsider the unconditional use of highly sensitive PII (like names or specific purchase histories) in broad-based campaigns. In situations where privacy concerns may be high (e.g., following public data breaches, or when marketing sensitive products), a less intrusive, context-based approach (like using location) may be significantly more effective and profitable. This suggests that “smarter,” not “creepier,” personalization is the optimal path. We recommend that firms and designers conduct A/B tests on different levels of personalization to find the sweet spot that maximizes relevance without activating a defensive response from their specific user base.

It is important to contextualize the significant interaction effect, which had a statistically small effect size. While small, such an effect can have considerable practical significance in a real-world marketing context. Digital advertising campaigns often operate at a massive scale, reaching millions of consumers. In such an environment, even a small percentage change in purchase intention can translate into substantial differences in revenue and customer retention. Our findings suggest that choosing an intrusive, PII-based personalization strategy over a moderate, contextual one could negatively impact thousands of customers, highlighting that what appears to be a small effect statistically can be highly meaningful from a business perspective.

### 5.4. Limitations and Future Research

This study has several limitations that offer avenues for future research.

First, as noted in the methods, our personalization manipulation has a conceptual confound between the quantity and type of data. While this design enhances ecological validity, future research should aim to deconstruct these components to isolate the unique impact of using PII versus simply using more data points.

Second, the news prime used to manipulate privacy concern may have triggered alternative psychological states, such as general negative affect or corporate distrust. Although our manipulation check confirmed the activation of our target construct, we did not measure these potential confounds. Future studies should co-measure these related constructs to isolate the unique variance attributable to situational privacy concern.

Third, our study operationalized situational privacy concern using a single prime (a news article). Future work could explore other contextual triggers, such as the platform on which an ad is seen (e.g., a trusted retail app vs. social media) or the type of product being advertised (e.g., a low-risk consumer good vs. a high-risk financial or health product), to build a richer model of situational privacy concerns.

Fourth, our findings may be culturally specific to our South Korean sample. Attitudes toward privacy are shaped by cultural dimensions such as individualism versus collectivism, which can influence where the ‘tipping point’ for personalization occurs. Cross-national research documents systematic differences in privacy concerns and ad acceptance (e.g., large-N comparisons across 57 countries and US vs. South Asia), implying the tipping point may shift with cultural context (Engström et al., 2023). We encourage replications in individualistic vs. collectivistic settings and with brand trust/ingroup–outgroup cues to map these boundaries.

Finally, our reliance on a hypothetical persona, while necessary for standardizing the context and maintaining rigorous experimental control, may not fully capture the realism of consumer responses. There is a clear trade-off between internal validity (achieved through control) and ecological validity (realism). A marketing message referencing one’s actual, personal purchase history is likely to evoke a more potent emotional and cognitive reaction than the simulated scenario used here. This limitation means our results should be interpreted as a conservative test of the backfire effect. To address these validity concerns, future research should employ field experiments. Collaborating with firms to conduct A/B tests on live marketing campaigns (Kohavi et al., 2020), using actual consumer data and measuring real behaviors (e.g., click-through rates, actual purchases), would provide invaluable evidence for the robustness of our findings in a natural setting.

## Figures and Tables

**Table 1:** Descriptive Statistics for Purchase Intention by Condition.

Privacy Concern	Personalization	Mean	N	Std. Deviation
High	High	5.58	62	0.90
	Medium	5.91	57	0.97
	Low	5.45	58	0.95
	Total	5.64	177	0.95
Low	High	6.43	63	0.83
	Medium	6.23	56	0.91
	Low	5.79	64	0.98
	Total	6.14	183	0.94
Total	High	6.01	125	0.96
	Medium	6.07	113	0.95
	Low	5.63	122	0.98
	Total	5.90	360	0.98

**Table 2:** ANOVA Results for the Effects of Personalization and Privacy Concern on Purchase Intention.

Source	Type III Sum of Squares	df	Mean Square	F	Sig.	Partial Eta Squared
Corrected Model	42.58	5	8.52	9.97	0.000	0.12
Intercept	12,490.14	1	12,490.14	14,622.35	0.000	0.98
Privacy Concern	22.64	1	22.64	26.50	0.000	0.07
Personalization	14.22	2	7.11	8.32	0.000	0.04
Privacy Concern * Personalization	5.48	2	2.74	3.21	0.042	0.02
Error	302.38	354	0.85			
Total	12,869.48	360				
Corrected Total	344.96	359				

## Data Availability

Data can be obtained upon request from the corresponding author.

## Appendix A. Experimental Stimuli

### Appendix A.1. Situational Privacy Concern Primes (News Articles)

**High-Concern Condition:**

“They See Everything in My Shopping Cart and Search History”… Google and Meta Make a Fortune with Deceptive Personalized Ads

Mr./Ms. A, an office worker in their 20s, recently searched Google twice for flight ticket prices for a trip to Thailand. To decide on a destination, they also searched for ‘#Bangkok’ on Instagram. Afterward, strangely enough, even when accessing work-related websites on Google, ads for Thailand flights from other airlines would appear, and on Instagram, an ad from a travel agency for a “7-night Thailand package tour for those in their 20s” popped up. Despite having searched only a few times, Google and Instagram seemed to know Mr./Ms. A all too well. The Personal Information Protection Commission has imposed a fine of approximately 100 billion won on Google and Meta (formerly Facebook) for violating the Personal Information Protection Act, the largest fine ever for such a violation. It was confirmed that the two companies had been collecting personal information for years without user consent to use for online personalized advertising. [22 September 2022, Newspaper A]

“Samsung Suffers Second Security Breach This Year After U.S. Customer Information Leak”

Samsung Electronics has experienced a customer data leak. According to a notice Samsung Electronics issued to its U.S. customers, in late July, an unauthorized third party breached some of Samsung’s U.S. systems and extracted information. In August, the company confirmed that the personal information of certain customers had been affected and issued the notice. According to Samsung Electronics’ announcement on the 2nd (U.S. local time), it is presumed that names, contact information, dates of birth, and product registration information were leaked. [5 September 2022, Newspaper B]

**Low-Concern (Control) Condition:**

“The Latest Interior Trend to Make Your Space the Most Comfortable Sanctuary”

As more consumers seek to decorate their spaces into comfortable sanctuaries, ‘Healing Minimalism’ is emerging as a new fall interior trend. Minimalism is an artistic and cultural movement that pursues simplicity and conciseness, and the formula for healing minimalism lies in this simplicity. It was recently emphasized that, “The mattress, which is responsible for the beginning and end of our day, is the piece of furniture that takes care of a modern person’s healing.” The article stressed, “Mattresses with simple designs equipped with functions faithful to the basics of sleep will provide consumers with a comfortable sanctuary.” [7 October 2022, Newspaper A]

“Bringing Autumn into Interior Design”

Within the “selective premium” trend, beds and sofas have become increasingly important items. This is because they not only support the body when lying or sitting down but also visually dictate the atmosphere of key spaces like the bedroom and living room. Various new products are being launched that capture the essence of autumn in interior furniture. In particular, they are elevating consumers’ interest and sense of interior design by using color palettes that match the fall and promote comfort. [19 September 2022, Newspaper B]

### Appendix A.2. Message Personalization Stimuli (Marketing Messages)

**Personalization**	**Message Text**
**High**	Hello, Mr./Ms. Kim Jung-ang. I see you’re near the Homeplus Sindorim branch, which you visited last week. We’re offering you a coupon for up to 20% off, based on the 100,000 won you recently spent at Homeplus! A fresh and delicious experience every day! Enjoy it at Homeplus.
**Medium**	Hello, customer. We’re offering a coupon for up to 20% off at Homeplus stores within 1km of your current location! A fresh and delicious experience every day! Enjoy it at Homeplus.
**Low**	Hello, customer. We’re offering a coupon for you to receive up to 20% off at Homeplus! A fresh and delicious experience every day! Enjoy it at Homeplus.
